# Baseline Monocyte Count Predicts Complete Response to Omalizumab in Chronic Spontaneous Urticaria: A Retrospective Analysis

**DOI:** 10.7759/cureus.100556

**Published:** 2026-01-01

**Authors:** İrem D Turhan, Berna Solak

**Affiliations:** 1 Dermatology, Sakarya University Training and Research Hospital, Sakarya, TUR; 2 Dermatology, Faculty of Medicine, Sakarya University, Sakarya, TUR

**Keywords:** basophil, biomarker, chronic spontaneous urticaria, inflammation, monocyte, omalizumab, treatment response

## Abstract

Introduction: Chronic spontaneous urticaria (CSU) is a distressing skin condition characterized by wheals and angioedema. While omalizumab is an effective biologic therapy for antihistamine-refractory CSU, a subset of patients shows partial or no response. Identifying reliable biomarkers to predict treatment outcomes remains a significant clinical need. This study aimed to investigate the relationship between systemic inflammatory parameters, specifically monocyte counts, and the clinical response to omalizumab.

Methods: This retrospective study included 52 patients with CSU treated with omalizumab (300 mg/four weeks) for at least 12 weeks at a tertiary referral center. Patients were stratified into two groups based on their response at week 12: "Complete Response" (Urticaria Activity Score over seven days (UAS7) = 0) and "Non-Complete Response." Baseline and post-treatment complete blood count (CBC) parameters, C-reactive protein (CRP), and total IgE levels were analyzed. Binary logistic regression was performed to identify independent predictors of response.

Results: Eleven patients (21.15%) achieved a complete response. The complete responder group exhibited significantly higher baseline median monocyte counts (0.68 vs. 0.40 K/µL, p = 0.001) and basophil counts (p = 0.032), but significantly lower baseline CRP levels (p = 0.003) compared to non-responders. Binary logistic regression analysis identified baseline monocyte count as the sole independent predictor of complete response (p = 0.036). Additionally, omalizumab treatment resulted in a significant reduction in neutrophil and monocyte counts, specifically in the responder group.

Conclusion: Higher baseline monocyte counts, alongside preserved basophils and low CRP levels, may define a distinct "responder clinical profile" (aligning with type I autoallergic CSU) that benefits maximally from omalizumab. Our findings suggest that higher baseline monocyte counts may serve as a potential independent predictor for complete treatment response.

## Introduction

Chronic spontaneous urticaria (CSU) manifests as a persistent dermatological condition defined by the recurrent appearance of evanescent wheals, angioedema, or a combination of both, lasting for longer than six weeks without an obvious external trigger [1]. This condition profoundly impacts the patient's quality of life, primarily due to severe pruritus and sleep disruption [2]. While the exact pathogenesis remains partially obscure, it is widely accepted that autoimmune pathways (type I and type IIb autoimmunity) and chronic inflammatory processes are the core drivers of the disease [3,4]. Omalizumab, a humanized recombinant monoclonal antibody targeting IgE, currently stands as the sole approved biologic agent for CSU patients who fail to respond to high-dose H1-antihistamines [1].

Although omalizumab demonstrates high efficacy in many cases, a non-negligible subset of patients (approximately 30-40%) exhibits either a partial response or no response at all [5]. Considering the substantial economic burden of biologic treatments and the impact of the disease, there is a critical clinical need to identify accessible biomarkers that can forecast treatment outcomes. Various hematological parameters derived from complete blood count (CBC), including mean platelet volume (MPV), neutrophil-to-lymphocyte ratio (NLR), and basophil counts, have been explored as potential markers in CSU [6-8]. Recently, the involvement of monocytes and macrophages in the inflammatory cascade of urticaria has garnered interest; however, their specific utility in predicting the therapeutic response to omalizumab has not been fully established [9].

In this retrospective analysis, our objective was to examine the alterations in hemogram and systemic inflammatory markers throughout the course of omalizumab therapy and to determine whether baseline values, specifically monocyte counts, could serve as predictive indicators for achieving a complete clinical response in resistant CSU cases.

## Materials and methods

Study design and population

We performed a retrospective chart review of patients managed at the Dermatology Outpatient Clinic of Sakarya University Training and Research Hospital. The study protocol complied with the ethical guidelines of the Declaration of Helsinki. Necessary ethical approval was granted by the Sakarya University Faculty of Medicine Non-Interventional Clinical Research Ethics Committee (Approval No.: E-71522473-050.04-372976-186). We screened the medical files of patients diagnosed with CSU who underwent omalizumab therapy between January 2020 and January 2024. Inclusion criteria mandated treatment with omalizumab (300 mg every four weeks) for a period of not less than 12 weeks and the availability of comprehensive medical datasets. Patients presenting with inducible urticaria, concurrent chronic inflammatory disorders, malignancies, parasitic infestations, or active infections were excluded to eliminate potential confounding factors affecting inflammatory markers.

Assessments and definitions

Pertinent data, including demographics, duration of disease, and family history, were extracted from the records. Disease activity was quantified using the Urticaria Activity Score over seven days (UAS7) [10] and the Urticaria Control Test (UCT) [11]. Laboratory investigations, comprising complete blood count (CBC), C-reactive protein (CRP), erythrocyte sedimentation rate (ESR), and total IgE levels, were documented at baseline (week 0) and following the 12th week of therapy.

Evaluation of treatment response

The clinical response was assessed at week 12 of treatment. "Complete Response" was rigorously defined as the attainment of a UAS7 score of 0 (complete symptom resolution). Patients failing to reach a UAS7 score of 0 were classified into the "Non-Complete Response" cohort.

Statistical analysis

Data analysis was conducted using Python version 3.x (Python Software Foundation, Wilmington, Delaware). The Kolmogorov-Smirnov test was utilized to assess the normality of the data distribution. Continuous variables were expressed as mean ± standard deviation or median (interquartile range, IQR), while categorical variables were reported as frequencies and percentages. For comparisons between independent groups, the Mann-Whitney U test was employed. The Wilcoxon signed-rank test was used to compare pre- and post-treatment values within groups. Categorical variables were compared using the chi-square test. Binary logistic regression analysis was executed to determine independent predictors of treatment outcomes. A p-value of <0.05 was deemed statistically significant.

## Results

Demographics and clinical characteristics

A total of 52 patients were included in the study. The mean age was 40.4 ± 14.2 years, and 40 (76.9%) were female. The median disease duration was four years (range: 1-20 years). A family history of urticaria was documented in seven (13.5%) patients. At week 12, 11 patients (21.15%) achieved a complete response (UAS7 = 0). There were no statistically significant differences between the responder and non-responder groups in terms of age, gender, BMI, or disease duration (p > 0.05) (Table 1).

**Table 1:** Demographic and clinical characteristics of patients stratified by treatment response at week 12. Data are presented as mean ± standard deviation, median (minimum-maximum), or number (percentage). Statistical tests used: The independent samples t-test was used for age (t-value indicated). Pearson's chi-square test was used for gender (χ² value indicated). Fisher’s exact test was used for categorical variables with low cell counts (indicated as F). The Mann-Whitney U test was used for non-normally distributed continuous variables (Z-scores indicated). SD: standard deviation; BMI: body mass index; UAS7: Urticaria Activity Score over seven days; UCT: Urticaria Control Test.

Parameter	Non-complete responder (n = 41)	Complete responder (n = 11)	Test value	p-value
Age (years), mean ± SD	38.8 ± 12.9	46.4 ± 17.8	t = -1.326	0.208
Gender, n (%)			χ² = 0.001	0.701
Female	32 (78.0%)	8 (72.7%)		
Male	9 (22.0%)	3 (27.3%)		
Disease duration (years), median (min-max)	4.0 (1.0 - 20.0)	2.0 (1.0 - 10.0)	Z = -1.534	0.125
BMI (kg/m²), median (min-max)	26.89 (15.43 - 54.77)	22.04 (18.71 - 30.85)	Z = -0.650	0.516
Family history of urticaria, n (%)	6 (14.6%)	1 (9.1%)	F	>0.999
Omalizumab dose escalation, n (%)			F	0.322
No	35 (85.4%)	11 (100.0%)		
Yes	6 (14.6%)	0 (0.0%)		
UAS7 score, median (min-max)				
Baseline	21.00 (5.00 - 42.00)	15.00 (4.00 - 35.00)	Z = -1.711	0.87
Week 12	6.00 (1.00 - 42.00)	0.00 (0.00 - 0.00)	Z = -5.421	<0.001
UCT score, median (min-max)				
Baseline	9.00 (2.00 - 14.00)	10.00 (4.00 - 14.00)	Z = -1.296	0.195
Week 12	14.00 (4.00 - 16.00)	16.00 (16.00 - 16.00)	Z = -4.852	<0.001

Comparison of baseline parameters

When baseline laboratory values were compared according to treatment response, the complete response group had significantly higher median monocyte counts (0.68 vs. 0.40 K/uL, p = 0.001) compared to the non-responder group. Additionally, responders exhibited higher (preserved) basophil counts (0.05 vs. 0.03 K/uL, p = 0.032). Conversely, baseline CRP levels were significantly lower in the complete response group (1.80 vs. 2.80 mg/L, p = 0.003).

Changes in parameters with treatment (week 0 vs. week 12)

In the complete response group, significant decreases were observed in monocyte counts (p = 0.010) at week 12 compared to baseline. Notably, neutrophil counts also showed a significant reduction in the responder group following omalizumab treatment (p = 0.041). Total IgE levels significantly increased in both groups at week 12, consistent with the expected pharmacodynamic effect of omalizumab (p < 0.05). Basophil counts did not change significantly with treatment in either group, although they remained higher in responders at week 12 (p = 0.049) (Table 2).

**Table 2:** Comparison of laboratory parameters between study groups and changes from baseline to week 12. Statistical tests used: P-value*: Comparison between groups (non-complete responder vs. complete responder) was performed using the Mann-Whitney U test (Z-scores indicated in parentheses). P-value**: Comparison between baseline and week 12 within each group was performed using the Wilcoxon signed-rank test (Z-scores indicated in parentheses). IQR: interquartile range; WBC: white blood cells; CRP: C-reactive protein; ESR: erythrocyte sedimentation rate.

Parameter	Non-complete responder (n = 41), Median (IQR)	Complete responder (n = 11), Median (IQR)	p-value* (between groups)
Total IgE (IU/mL)			
Baseline	137.0 (18.0 - 1190.0)	120.0 (40.0 - 619.0)	0.893 (Z=-0.14)
Week 12	441.5 (17.0 - 2680.0)	338.0 (131.0 - 2020.0)	0.991 (Z=-0.01)
p-value** (within group)	<0.001 (Z=-3.89)	0.010 (Z=-2.58)	
Neutrophil count (K/µL)			
Baseline	4.30 (1.90 - 9.80)	5.10 (3.50 - 10.20)	0.086 (Z=-1.72)
Week 12	4.40 (1.85 - 8.40)	3.50 (2.59 - 6.40)	0.078 (Z=-1.76)
p-value** (within group)	0.120 (Z=-1.56)	0.041 (Z=-2.04)	
Basophil count (K/µL)			
Baseline	0.03 (0.00 - 0.09)	0.05 (0.01 - 0.14)	0.032 (Z=-2.14)
Week 12	0.03 (0.00 - 0.09)	0.06 (0.02 - 0.13)	0.049 (Z=-1.97)
p-value** (within group)	0.371 (Z=-0.90)	0.754 (Z=-0.31)	
Eosinophil count (K/µL)			
Baseline	0.15 (0.05 - 1.00)	0.14 (0.00 - 0.60)	0.583 (Z=-0.55)
Week 12	0.15 (0.04 - 0.56)	0.21 (0.00 - 0.33)	0.340 (Z=-0.95)
p-value** (within group)	0.264 (Z=-1.12)	0.594 (Z=-0.53)	
Lymphocyte count (K/µL)			
Baseline	2.30 (0.00 - 3.96)	2.30 (0.51 - 3.50)	0.982 (Z=-0.02)
Week 12	2.50 (0.80 - 4.75)	2.30 (1.50 - 4.20)	0.875 (Z=-0.16)
p-value** (within group)	0.545 (Z=-0.61)	0.152 (Z=-1.43)	
Monocyte count (K/µL)			
Baseline	0.40 (0.20 - 0.75)	0.68 (0.36 - 1.10)	0.001 (Z=-3.29)
Week 12	0.40 (0.23 - 0.90)	0.52 (0.26 - 0.79)	0.063 (Z=-1.86)
p-value** (within group)	0.561 (Z=-0.58)	0.010 (Z=-2.58)	
WBC count (K/µL)			
Baseline	7.40 (4.20 - 14.10)	8.00 (6.70 - 14.90)	0.209 (Z=-1.26)
Week 12	7.80 (4.40 - 11.80)	6.40 (4.90 - 11.10)	0.190 (Z=-1.31)
p-value** (within group)	0.113 (Z=-1.59)	0.155 (Z=-1.42)	
Platelet count (K/µL)			
Baseline	284.0 (3.13 - 512.0)	296.0 (213.0 - 545.0)	0.494 (Z=-0.68)
Week 12	287.0 (168.0 - 434.0)	299.0 (172.0 - 659.0)	0.840 (Z=-0.20)
p-value** (within group)	0.659 (Z=-0.44)	0.656 (Z=-0.45)	
CRP (mg/L)			
Baseline	2.80 (1.40 - 16.50)	1.80 (1.10 - 6.30)	0.003 (Z=-2.97)
Week 12	2.30 (1.00 - 22.00)	1.60 (1.30 - 3.40)	0.068 (Z=-1.83)
p-value** (within group)	0.037 (Z=-2.09)	0.540 (Z=-0.61)	
ESR (mm/h)			
Baseline	12.00 (3.00 - 51.00)	15.00 (1.00 - 48.00)	0.119 (Z=-1.56)
Week 12	10.50 (3.00 - 34.00)	15.50 (1.00 - 24.00)	0.662 (Z=-0.44)
p-value** (within group)	0.600 (Z=-0.52)	0.405 (Z=-0.83)	

Predictors of response

Binary logistic regression analysis was performed to evaluate the predictors of complete response (Table 3). The model included baseline UAS7, CRP, basophil count, and monocyte count. Baseline monocyte count was identified as the only independent predictor of complete response to omalizumab (p = 0.036), although with a wide confidence interval likely due to the small sample size.

**Table 3:** Binary logistic regression analysis for independent predictors of complete response to omalizumab. B: regression coefficient; S.E.: standard error; OR: odds ratio; CI: confidence interval; UAS7: Urticaria Activity Score over seven days; CRP: C-reactive protein. Model evaluation: Hosmer and Lemeshow test, p = 0.831; Nagelkerke R-square = 0.422.

Variable (baseline)	B	S.E.	p-value	OR (Exp(B))	95% CI for OR
					Lower - Upper
UAS7 score	-0.045	0.041	0.271	0.956	0.883 - 1.036
CRP level	-0.313	0.263	0.234	0.731	0.437 - 1.225
Basophil count	5.349	18.408	0.771	210.355	0.000 - 98111.0
Monocyte count	6.621	3.154	0.036	750.665	1.553 - 362853.1
Constant	-3.255	1.815	0.073	0.039	-

## Discussion

In this study, we investigated the relationship between systemic inflammatory parameters and the clinical response to omalizumab in patients with resistant CSU. Our analysis yielded several clinically relevant findings. Most notably, we identified that a higher baseline monocyte count is an independent predictor of achieving a complete response (UAS7 = 0). Furthermore, our results regarding basophils and CRP provide significant insights into the different clinical profiles of CSU and their responsiveness to anti-IgE therapy. We strictly defined "complete response" as UAS7 = 0 to identify patients with total symptom clearance, distinguishing them from those with merely improved but residual disease, which aligns with the "treat-to-target" approach in CSU management.

The predictive value of monocytes is a relatively novel finding in the context of omalizumab treatment. Monocytes are key players in chronic inflammation and have been shown to express the high-affinity IgE receptor FcεRI in allergic conditions, with increased FcεRI expression on monocytes from atopic individuals and the ability of peripheral monocytes to bind to monomeric IgE via FcεRI [12,13]. Previous studies have demonstrated that peripheral blood monocytes in CSU patients are activated and exhibit significantly increased expression of tissue factor (TF), which triggers the extrinsic coagulation cascade and contributes to vascular permeability and wheal formation [9]. This suggests that monocytes are not merely bystanders but active contributors to the disease pathophysiology. In our study, the "complete responder" group had higher baseline monocyte counts, suggesting a predominant monocyte-associated inflammatory profile. The significant reduction in monocyte counts following omalizumab treatment suggests that the drug may mitigate this pro-coagulant state by dampening monocyte activation, thereby resolving the symptoms.

Our findings are consistent with the currently proposed concept of two distinct CSU endotypes: "autoallergic" (type I) and "autoimmune" (type IIb) [3,4]. The "non-responder" group in our study exhibited significantly lower baseline basophil counts (basopenia) and higher CRP levels. This profile aligns with the type IIb autoimmune profile, which is driven by IgG autoantibodies against IgE or FcεRI and is generally known to be more refractory to antihistamines and omalizumab [3]. Indeed, basophils in these patients are known to be recruited into the skin lesions or desensitized, leading to reduced peripheral counts [14]. In contrast, the "complete responder" group was characterized by higher baseline basophils and lower CRP levels, consistent with a type I autoallergic profile. We hypothesize that the higher baseline monocyte count observed in our study constitutes an integral part of this "good responder" subgroup. This implies that a distinct immune profile, characterized by higher (preserved) basophils, higher monocytes, and low systemic inflammation, marks the ideal candidate for rapid and complete remission with omalizumab.

Interestingly, although total IgE levels increased significantly in all patients post-treatment, a reliable marker of drug adherence and pharmacodynamics [15], we found no significant difference in baseline total IgE levels between responders and non-responders. While some studies suggest high baseline IgE predicts better response [16], our data indicate that the baseline serum IgE level alone may not be a sufficient discriminator for treatment outcome. This reinforces the idea that omalizumab’s efficacy involves complex cellular mechanisms, such as the downregulation of FcεRI on mast cells and basophils, rather than solely the sequestration of free serum IgE [17].

Although the difference did not reach statistical significance, we observed a notable trend regarding body mass index (BMI). The median BMI was markedly lower in complete responders (22.04 kg/m²) compared to non-responders (26.89 kg/m²). Obesity is characterized by a state of chronic low-grade inflammation, with adipose tissue secreting pro-inflammatory cytokines like IL-6 and tumor necrosis factor-alpha, which may exacerbate urticarial inflammation [18]. It has been hypothesized that higher body mass may influence the effective exposure to fixed-dose biologic therapies. This observation suggests that weight management could be a supportive strategy in resistant cases.

Beyond clinical symptom control, we observed a significant reduction in neutrophil counts following omalizumab treatment in the responder group. Although CSU has traditionally been regarded as a mast cell-driven disease, increasing evidence indicates that neutrophils also contribute to the inflammatory cascade through the release of proteolytic enzymes, reactive oxygen species, and pro-inflammatory mediators [1]. Recent reviews have highlighted that omalizumab treatment may be associated with reductions in neutrophil-related inflammatory parameters, such as neutrophil counts and neutrophil-derived ratios, reflecting a broader immunomodulatory effect beyond mast cell stabilization [19]. Our findings provide supportive clinical observations for this concept, suggesting that effective anti-IgE therapy may attenuate systemic inflammatory activity, including neutrophil involvement, particularly in patients achieving complete clinical remission. However, given the observational nature of the available data, the precise role of neutrophils in mediating treatment response warrants further investigation in prospective studies.

The primary limitations of our study are its retrospective design and the relatively small sample size, which resulted in wide confidence intervals in the regression analysis. Additionally, defining "complete response" strictly as UAS7 = 0 yielded a smaller responder group, though this ensured a clear distinction between residual disease and complete healing. Further prospective studies with larger cohorts are needed to validate the cut-off values for monocyte counts.

## Conclusions

In conclusion, this study highlights the potential of baseline monocyte count as a novel, inexpensive, and easily accessible biomarker for predicting a complete clinical response to omalizumab in patients with resistant CSU. Our findings suggest that patients with higher monocyte counts, preserved basophils, and lower systemic inflammation (low CRP) represent a distinct clinical profile that benefits maximally from anti-IgE therapy.
